# Low-Grade Glioma in the Differential Diagnosis of Limbic Encephalitis

**DOI:** 10.7759/cureus.83899

**Published:** 2025-05-11

**Authors:** Eliezer Villanueva-Castro, Rebeca Hernández Reséndiz, Marco Antonio Munuzuri-Camacho, Domingo J Coutinho Thomas, Luz de Alicia Jiménez-Quintero, Bernardo Cacho-Díaz, Ignacio Reyes-Moreno, Guillermo A Gutierrez-Aceves, Vicente Guerrero-Juarez, Jesus Ramirez-Bermudez, Alberto González-Aguilar

**Affiliations:** 1 Department of Neurosurgery, Instituto Nacional de Neurología y Neurocirugía Manuel Velasco Suárez, Mexico City, MEX; 2 Department of Neurology, Hospital Angeles Universidad, Mexico City, MEX; 3 Department of Neuro-Oncology, International Cancer Center, Mexico City, MEX; 4 Department of Neuro-Oncology, National Institute of Cancer, Mexico City, MEX; 5 Department of Neuro-Oncology, The American British Cowdray Medical Center, Mexico City, MEX; 6 Department of Neuroradiosurgery, Instituto Nacional de Neurología y Neurocirugía Manuel Velasco Suárez, Mexico City, MEX; 7 Department of Psychiatry, Instituto Nacional de Neurología y Neurocirugía Manuel Velasco Suárez, Mexico City, MEX

**Keywords:** diagnosis, limbic encephalitis, low grade gliomas, neurology, neurosurgery

## Abstract

Background: Limbic encephalitis (LE) is an inflammatory syndrome affecting the limbic system, often presenting with seizures, memory impairment, and behavioral disturbances. While autoimmune and infectious causes are common, gliomas can mimic this condition, leading to diagnostic challenges.

Methods: A retrospective review of glioma cases initially diagnosed as LE was conducted at two centers in Mexico (The American British Cowdray and National Institute of Neurology and Neurosurgery) from 2010 to 2022. Inclusion criteria included acute clinical presentation, magnetic resonance imaging (MRI) T2 hyperintensities in the limbic system, lumbar puncture, paraneoplastic panel, cerebrospinal fluid (CSF) viral studies, and histopathological confirmation. Patients with infectious, autoimmune, or other identified etiologies were excluded.

Results: Nine patients (mean age 45.2 years; six males, three females) met the criteria. Initial presentations included seizures (5/9), encephalopathy (5/9), fever (1/9), and psychotic symptoms (2/9). MRI revealed limbic system hyperintensities with minimal contrast enhancement. Spectroscopy and perfusion MRI findings suggested low-grade gliomas (LGGs). Histopathology confirmed astrocytoma (5/9), oligoastrocytoma (3/9), and oligodendroglioma (1/9). All patients received steroids, and some received additional therapies (plasma exchange (PLEX), acyclovir, immunoglobulin).

Conclusions: Gliomas can present as LE, leading to potential misdiagnosis. Advanced imaging and histopathological confirmation are essential for accurate differentiation, ensuring appropriate treatment and improved patient outcomes.

## Introduction

Limbic encephalitis (LE) is an inflammatory condition involving the limbic system, presenting neurological symptoms such as seizures, short-term memory loss, catatonia, and behavioral disturbances. It is typically subacute and progressive. One of the most important aspects is the hypersignal in the mesial temporal lobe (limbic system), which is not present in all cases, but its presence necessitates an approach such as LE [1,2].

Clinical features of supratentorial tumors vary according to their anatomic location, biological aggressiveness, and patient age. They can be either completely asymptomatic or present with signs of raised intracranial pressure, seizures (about 40% of cases), behavioral changes, speech disorders, declining school performance, or hemiparesis. Gliomas are the most common primary brain tumors. These tumors are highly aggressive with a dismal prognosis despite treatment, making early diagnosis fundamental. Typical glioma cases present with indolent symptoms over months that progressively worsen, leading to emergency consultation [3]. However, grade 2 gliomas are slow-growing neoplasms with distinct phases: asymptomatic, clinical onset, anaplastic degeneration, and refractory/progression [4]. Due to the variety of clinical presentations, glioma diagnosis can be challenging.

This study aims to present a retrospective case series of gliomas initially misdiagnosed as LE and to examine the clinical, imaging, and histopathological features that contributed to this diagnostic confusion. To our knowledge, the phenomenon of gliomas mimicking LE remains underreported in the literature, and there is limited guidance on distinguishing features. The misdiagnosis of gliomas as LE can result in significant delays in appropriate oncologic treatment, which has serious clinical consequences. Our goal is to raise awareness of gliomas as a critical differential diagnosis in patients presenting with LE-like features.

In order to investigate this diagnostic overlap, we retrospectively reviewed cases from two tertiary referral centers in Mexico between 2010 and 2022, selected based on the availability of complete clinical, radiological, and histopathological data. Here, we present a clinical series of gliomas that were initially approached as LE due to their presentation and neuroimaging findings, highlighting glioma as a differential diagnosis in this syndrome.

## Materials and methods

A retrospective review of LE caused by glioma was conducted at two different centers in Mexico (American British Cowdray Hospital and the National Institute of Neurology and Neurosurgery). Neuro-oncology databases from both centers were reviewed for the period 2010-2022. Due to the nature of the databases and variations in diagnostic labeling, the exact number of initial LE cases screened and excluded could not be determined. However, only cases with complete clinical records and confirmed histopathological diagnoses were included to ensure diagnostic certainty.

Inclusion criteria were (1) acute clinical presentation suggestive of LE, (2) T2 hyperintensities on magnetic resonance imaging (MRI) involving the limbic system, (3) lumbar puncture performed, (4) testing for viral pathogens in cerebrospinal fluid (CSF), (5) paraneoplastic and autoimmune antibody panel testing, and (6) histopathological confirmation of glioma. Patients with other identifiable infectious, autoimmune, or metabolic causes were excluded.

Two reviewers (trained physicians with experience in neuro-oncology and retrospective clinical research) independently performed the chart review using a standardized data extraction protocol. This protocol included predefined variables such as demographic data, initial clinical presentation, neuroimaging characteristics, CSF parameters (protein, glucose, and cell count), electroencephalography (EEG) findings, results from autoimmune/paraneoplastic panels, and final histopathological diagnosis. Any discrepancies between reviewers were resolved by consensus. The use of structured data collection forms helped ensure consistency and reproducibility of the extraction process.

Not all patients underwent advanced imaging modalities (MR spectroscopy or perfusion MRI), primarily due to differences in availability between institutions. MRI protocols were not standardized across centers. Autoimmune LE was assessed based on the most current clinical and diagnostic consensus criteria available at the time of diagnosis, and all cases underwent testing with commercial paraneoplastic and autoimmune antibody panels (including anti-NMDAR, LGI1, CASPR2, Hu, Yo, Ri, and Ma2, among others).

Descriptive statistical analyses were conducted, including means, medians, and ranges for continuous variables and frequencies for categorical variables. Abnormal CSF values were defined as protein >45 mg/dL, glucose <40 mg/dL or <50% of serum glucose, and cell count >5 WBC/µL. No inferential statistics were performed due to the small sample size. Data collection was anonymized in accordance with ethical standards, and the study was approved by the institutional review board under protocol number 77/23.

## Results

**Figure 1:**
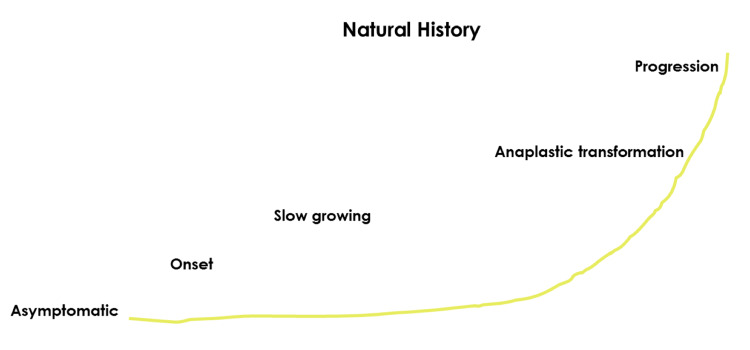
Natural history of low-grade gliomas A schematic representation of the progression of low-grade gliomas, illustrating the different phases of tumor development. The timeline starts with an asymptomatic phase, followed by clinical onset and a slow-growing phase. Eventually, anaplastic transformation occurs, leading to rapid tumor progression (This image is original and was developed by the authors of this article)

**Figure 2:**
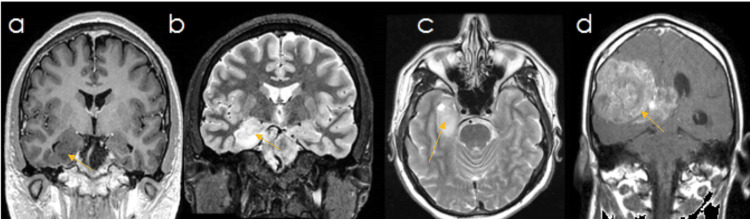
Radiological progression in patient 2 MRI: magnetic resonance imaging; FLAIR: fluid-attenuated inversion recovery Radiological progression in a two-month follow-up in case 2: (A) T1-weighted coronal MRI with hypointense lesion pointed in yellow. (B) T2-weighted coronal MRI with a hyperintense lesion pointed in yellow. (C) T2-weighted axial MRI with a hyperintense lesion pointed in yellow. (D) T2-weighted FLAIR axial MRI with a hyperintense diffuse lesion pointed in yellow

This retrospective study focused on analyzing the progression of low-grade gliomas (LGGs) and their relationship with LE. The natural history of LGGs can be predictable, as mentioned before (Figure 1), and can be recorded via neuroimaging, as it was in patient 2 (Figure 2).

**Figure 3:**
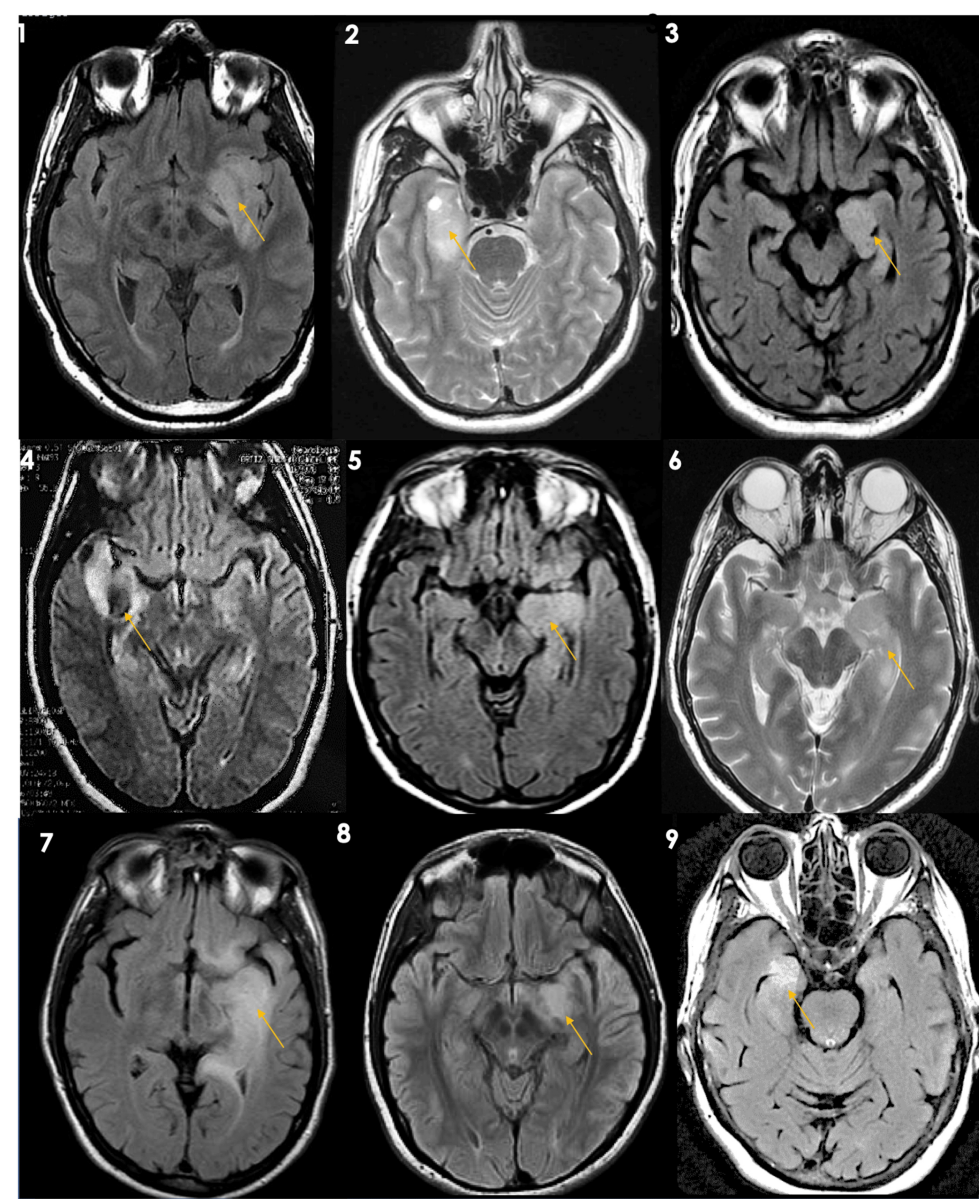
MRI series of the nine patients MRI: magnetic resonance imaging MRI series of the nine patients from 1 to 9, respectively, presenting unilateral hyperintense diffuse lesions pointed in yellow

A total of nine patients with LE fulfilled the criteria. The mean age was 45.2 years, with 6/9 patients being male and 3/9 female. The presentation at diagnosis consisted of new-onset seizures in 5/9 patients (including two with status epilepticus), encephalopathy in 5/9, fever in 1/9, and psychotic symptoms in 2/9. All patients met possible criteria for autoimmune disease. Short-term memory loss (STML) was present in 7/9 patients, but epileptic activity was the most significant sign prompting medical attention. Neuroimaging showed hypersignal in the limbic system in all cases, which was unilateral, with a very discrete mass effect and minimal or no contrast enhancement (Figure 3).

**Figure 4:**
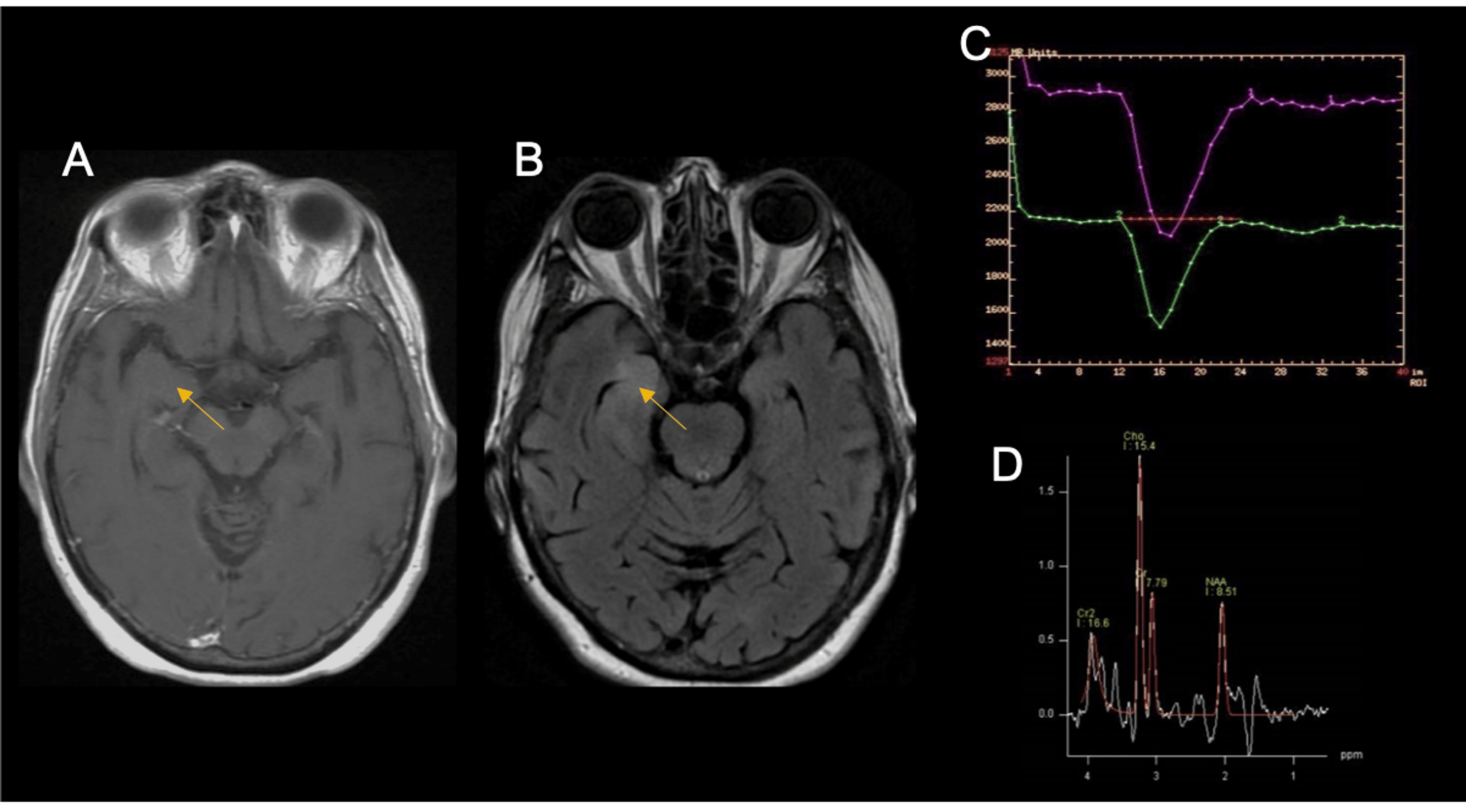
MR spectroscopy findings in patient 9 MRI: magnetic resonance imaging; FLAIR: fluid-attenuated inversion recovery; Cho: choline-containing compounds; NAA: N-acetyl-aspartate; (A) T2-weighted axial MRI with a discreet hyperintense lesion pointed in yellow. (B) T2 FLAIR-weighted MRI with a unilateral hyperintense lesion pointed in yellow. (C) Increased cerebral blood volume 1.9 times higher compared to baseline, 1.7 is the cutoff point (purple vs green, respectively, and the cutoff point is represented in red).  (D) Choline peak relative to N-acetyl aspartate (labeled as Cho and NAA, respectively), suggesting a tumor etiology

**Figure 5:**
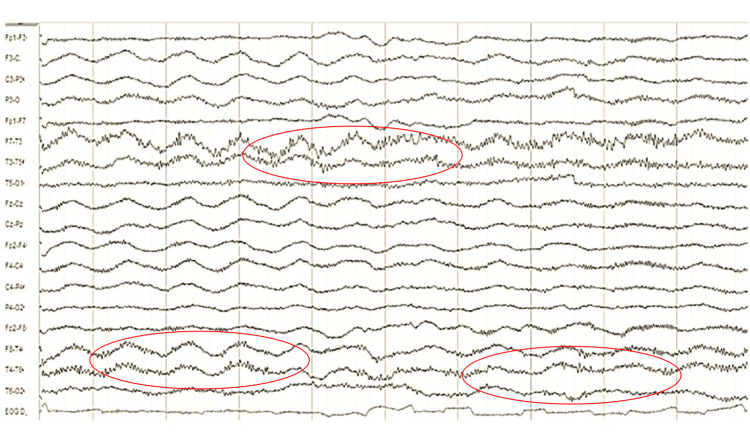
EEG of patient 6 EEG: electroencephalography; NMDA: N-methyl-D-aspartate EEG shows extreme delta brush, a distinctive pattern characterized by generalized delta activity (1 to 1.5 Hz) with superimposed fast beta-range rhythms, typically associated with anti-NMDA receptor encephalitis (circled in red)

**Figure 6:**
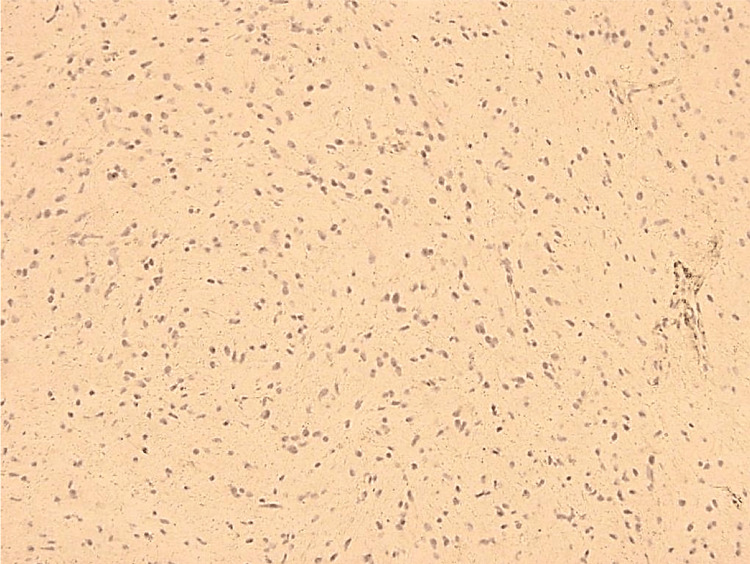
Histopathological analysis of patient 3 Histopathological analysis of patient 3 shows a grade 2 astrocytoma displaying mild cellularity with moderate nuclear atypia and low mitotic activity and a diffuse infiltrative pattern without necrosis. The tumor cells are positive for glial fibrillary acidic protein (GFAP), indicating their astrocytic origin

Spectroscopy results were available for eight cases, showing a mean Cho/NAA ratio of 2.3 and Cho/Cr of 1.7. Perfusion MRI was available for five cases, with a mean relative cerebral blood volume (rCBV) of 1.6. Figure 4 represents the MR spectroscopy findings in patient 9. Lumbar puncture and EEG findings are described in Table 1. Patient 6 had an EEG with extreme delta brush, as shown in Figure 5. Histopathological diagnoses included astrocytoma (5/9), oligoastrocytoma (3/9), and oligodendroglioma (1/9), all classified as grade 2 gliomas (Figure 6). All patients received steroids and other treatments (three patients underwent plasma exchange (PLEX), four received acyclovir, and one received immunoglobulin), with clinical response observed in all cases (Table 1). 

**Table 1:** Presentation, clinical characteristics, treatment, and outcome in the nine patients included in the study CE: cerebral encephalitis; STML: short-term memory loss; CSF: cerebrospinal fluid; Prot: protein; Gluc CSF: glucose in cerebrospinal fluid; EEG: electroencephalography; PLEDs: periodic lateralized epileptiform discharges-repetitive, focal EEG abnormalities often associated with acute cerebral injury; Cho: choline-containing compounds; NAA: N-acetyl-aspartate; Cr: creatinine Data outlining clinical characteristics, treatment, follow-up, test results, and patient evolution during hospitalization, as well as final outcomes

n	Age (years)	CE	STML	Onset clinical features	CSF aspect	Prot CSF mg/dL	Gluc CSF mg/dL	Cells (WBC/ul)	EEG	Cho/NAA	Cho/Cr	rCBV	Autoimmune criteria	Admission diagnosis	Final diagnosis	Treatment	Outcome
1	38	No	Yes	Psychotic syndrome	Normal	90	Low	12	PLEDs	3.6	1.3	ND	Possible	Viral encephalitis	Oligoastrocytoma 2	Steroid, acyclovir	Response
2	36	No	Yes	Complex partial seizure and fever	Normal	30	Normal	7	Slow	4.9	3.3	2.2	Possible	Viral encephalitis	Oligoastrocytoma 2	Steroid, acyclovir	Response
3	65	No	Yes	Epilepticus status	Normal	80	Elevated	6	PLEDs	1.5	1.8	ND	Possible	Autoimmune encephalitis	Astrocytoma 2	Steroid and PLEX	Response
4	57	No	Yes	Encephalopathy	Normal	67	Elevated	8	Epileptic activity	1.6	2	ND	Possible	Viral encephalitis	Astrocytoma 2	Steroid, acyclovir	Response
5	42	No	No	Complex seizures, encephalopathy	Normal	50	Normal	2	Epileptic activity	2.5	1.2	1.1	Possible	Autoimmune encephalitis	Oligodendroglioma 2	Steroid, PLEX	Response
6	49	No	Yes	Psychotic and encephalopathy	Normal	35	Normal	7	Delta-brush	1.8	2	1.2	Possible	Autoimmune encephalitis	Astrocytoma 2	Steroid	Response
7	35	No	Yes	Epilepticus status	Normal	48	Normal	4	Epileptic activity	1.5	1.4	1.8	Possible	Autoimmune encephalitis	Oligoastrocytoma 2	Steroid, IgV	Response
8	40	No	Yes	Complex seizures, encephalopathy	Normal	53	Normal	9	Epileptic activity	ND	ND	ND	Possible	Autoimmune encephalitis	Astrocytoma 2	Steroid, PLEX	Response
9	45	No	No	Encephalopathy and seizures	Normal	55	Elevated	6	PLEDs	1.5	1.2	1.9	Possible	Viral encephalitis	Astrocytoma 2	Steroid, acyclovir	Response

## Discussion

LE is a subacute syndrome commonly caused by autoimmune disorders, paraneoplastic syndromes, neuroinfectious conditions, and, in rare cases, malignant neoplasms. The clinical diagnosis is often difficult due to symptom overlap with other neuropsychiatric entities. Conventional imaging may struggle to distinguish encephalitis from glial tumors, especially low-grade malignancies such as oligodendroglioma or oligoastrocytoma [5].

This series highlights the importance of considering infiltrative gliomas in patients presenting with doubtful or uncommon imaging features of LE. In this study, nine cases with acute neurological symptoms and no prior illness history were initially approached as LE due to limbic system involvement in neuroimaging. It is well known that 10-40% of supratentorial tumors manifest with seizures as the sole symptom, with most diagnosed as LGGs [6]. The temporal lobe is the most common location for LGGs, often causing complex partial seizures [7,8]. The association between seizures and such tumors has been a focus of epileptologists and epilepsy surgeons since Hughlings Jackson’s report in the 1880s [9].

Radiologically, nearly all tumors were hypointense on T1-weighted and hyperintense on T2-weighted and fluid-attenuated inversion recovery (FLAIR) MRI sequences. Most cases involved only mesial structures (31/58.5%). No series have reported gliomas mimicking LE, though individual cases exist. Lumbar punctures are rarely performed in these patients due to significant mass effect, but our series showed CSF pleocytosis, leading to an initial misclassification as an inflammatory or infectious process. Treatment with steroids and acyclovir was undertaken. These findings can be explained by: (a) underestimated leptomeningeal infiltration by glioma (reported in 25% of cases), and (b) elevated CSF protein levels after seizures, observed in 60.9% of cases, particularly in older male patients with generalized seizures [10].

MR spectroscopy findings typical of brain neoplasms include decreased N-acetyl aspartate (NAA), increased choline (Cho), and decreased creatine (Cr). Non-neoplastic CNS processes, including infections, can produce similar MR spectroscopy profiles [11]. The diagnostic accuracy of MR spectroscopy varies, with a reported sensitivity of 0.95 and specificity of 1.0 in non-blinded assessments, dropping to 0.88 and 0.80 in blinded evaluations [12]. Perfusion MRI is useful for evaluating vascularity, with rCBV values between 1 and 2 indicating LGG [13]. Differentiating neoplastic from non-neoplastic lesions remains challenging, as no consensus exists for LGG cut-off values [14,15].

This series demonstrates the diagnostic difficulty in acutely ill neurological patients with limbic system involvement. Currently, autoimmune encephalitis is frequently diagnosed, but this syndrome may be overdiagnosed, potentially delaying glioma detection. Although autoimmune causes must be considered, LGGs should also be included in the differential diagnosis. This study underscores the importance of a multidisciplinary approach and close follow-up to ensure accurate diagnosis and timely intervention [16,17].

Limitations

This study has several important limitations. First, due to its retrospective design and the lack of a centralized database, it was not possible to determine the total number of LE cases reviewed or excluded, which limits transparency in the selection process and may introduce selection bias. Additionally, the diagnostic workup was not fully standardized, as advanced imaging studies such as MR spectroscopy or perfusion MRI were not available for all patients due to institutional limitations, which could have affected the homogeneity of the findings.

Second, while autoimmune encephalitis criteria were applied based on the most updated international guidelines, the specific antibody panels varied depending on availability at each center, which may have influenced the accuracy of classification and the potential for misdiagnosis. This highlights the variability in diagnostic approaches and the limitations of the context in which the study was conducted.

Moreover, the small sample size, although reflecting the rarity of this clinical presentation, limits the ability to generalize the findings to a broader population. The study included only nine cases over a 12-year period, restricting its inferential value and the ability to draw conclusions about the prevalence or clinical features of LGGs that mimic LE.

Finally, the lack of consensus on rCBV thresholds for LGGs limits the diagnostic certainty in perfusion imaging. Additionally, the overlap with inflammatory findings, such as CSF pleocytosis and steroid responsiveness, can mislead clinicians into mistakenly diagnosing autoimmune encephalitis. These factors underscore the diagnostic complexity and highlight the need for multidisciplinary evaluation and longitudinal follow-up to ensure accurate and timely diagnosis.

## Conclusions

LE is a complex syndrome with diverse etiologies, including autoimmune, infectious, and neoplastic causes. This study highlights the diagnostic challenge of LGGs mimicking LE due to their acute presentation and involvement of the limbic system on MRI, which can delay the diagnosis and treatment of gliomas. Although MR spectroscopy and perfusion MRI can aid in differentiation, histopathological confirmation remains essential. Overdiagnosis of autoimmune encephalitis may lead to unnecessary treatments and delay oncological management.

We acknowledge the importance of balancing diagnostic testing with clinical suspicion. In cases with overlapping symptoms with autoimmune encephalitis, it is crucial to avoid unnecessary testing. This study emphasizes the need for careful clinical judgment and multidisciplinary collaboration to perform appropriate tests, avoiding misdiagnosis and ensuring timely intervention.

We agree that the study does not include a matched cohort of autoimmune encephalitis cases for comparison, nor does it quantify the delay in diagnosis. This is a limitation, and while we hypothesize that autoimmune encephalitis may be overdiagnosed, leading to a delay in tumor treatment, we understand that this cannot be conclusively supported by the data presented. We have revised the manuscript to frame this more clearly as a hypothesis, based on the observed patterns of misdiagnosis in our study, rather than a definitive conclusion. A multidisciplinary approach is essential in recognizing gliomas as a differential diagnosis in LE, ensuring proper management and potentially improving patient outcomes.
